# Supplementary material for: Free-moving Quantitative Gamma-ray Imaging
Source: Sci Rep. 2021 Oct 15;11:20515. doi: 10.1038/s41598-021-99588-z (PMC8519933; doi:10.1038/s41598-021-99588-z)
Supplement: Supplementary file 1 — Supplementary Figures. [file 41598_2021_99588_MOESM1_ESM.pdf]

Supplementary Information
Free-moving Quantitative Gamma-ray Imaging

Daniel Hellfeld[1,*], Mark S. Bandstra[1], Jayson R. Vavrek[1], Donald L. Gunter[2], Joseph C. Curtis[1], Marco Salathe[1], Ryan Pavlovsky[1], Victor Negut[1], Paul J. Barton[1], Joshua W. Cates[1], Brian J. Quiter[1], Reynold J. Cooper[1], Kai Vetter[1, 3], Tenzing H. Y. Joshi[1]

[1]Nuclear Science Division, Lawrence Berkeley National Laboratory, Berkeley, CA 94720 USA, [2]Gunter Physics, Inc., Lisle, IL 60532 USA, [3]Department of Nuclear Engineering, University of California, Berkeley, Berkeley, CA 94720 USA

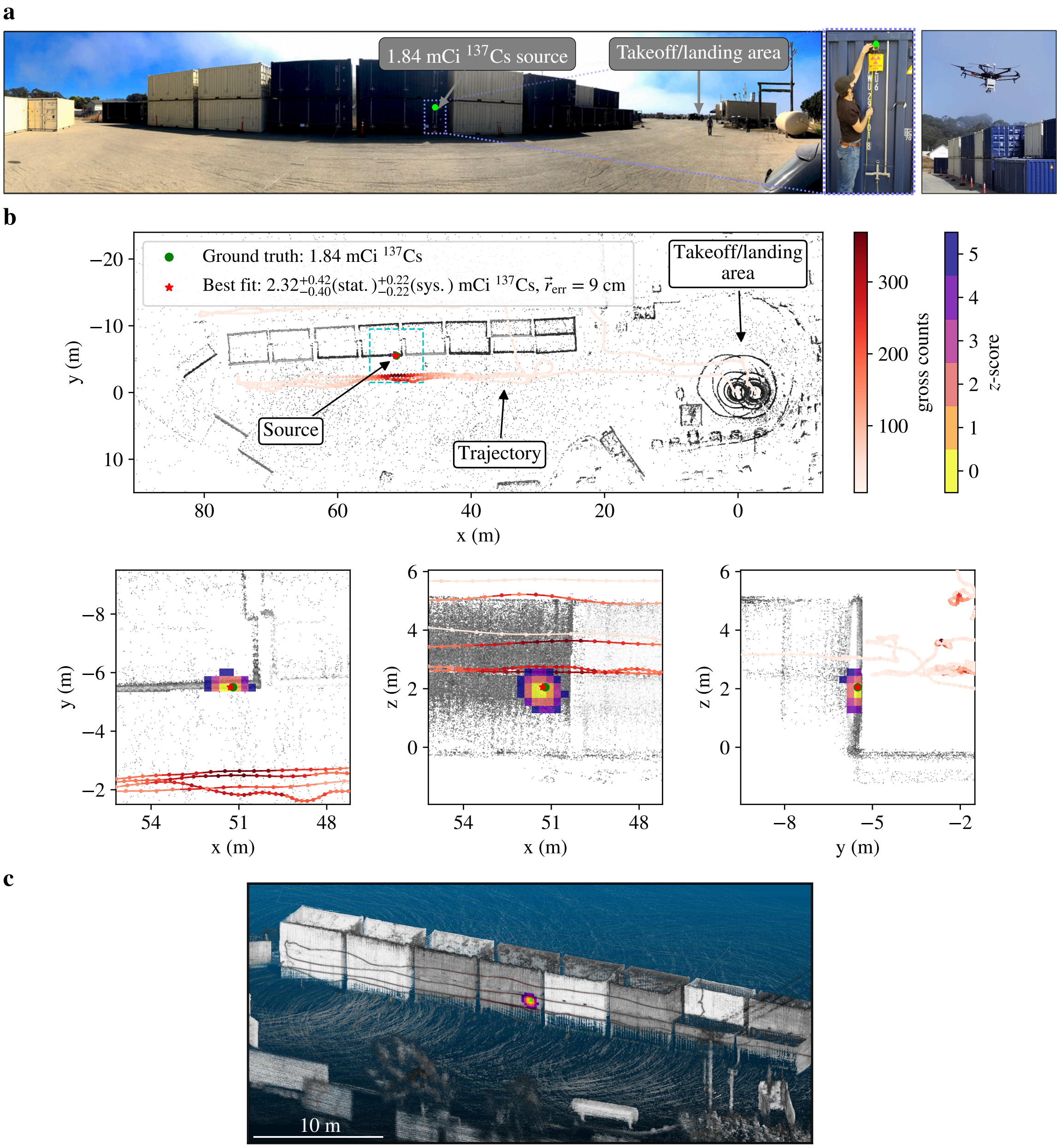

Figure S1. **Point-source reconstruction results**. (**a**) Point-source scenario in which a 1.84 mCi ^{137}Cs source was placed ≈2.5 m above the ground on the exterior of a cargo container stack. MiniPRISM was flown remotely on a sUAS and surveyed the stack in ≈7 min. (**b**) Quantitative Compton PSL reconstruction following the survey of the cargo containers, including a full top-down projection as well as three zoomed-in projections near the true source location. (**c**) Rendering of the colorized 3D point cloud.

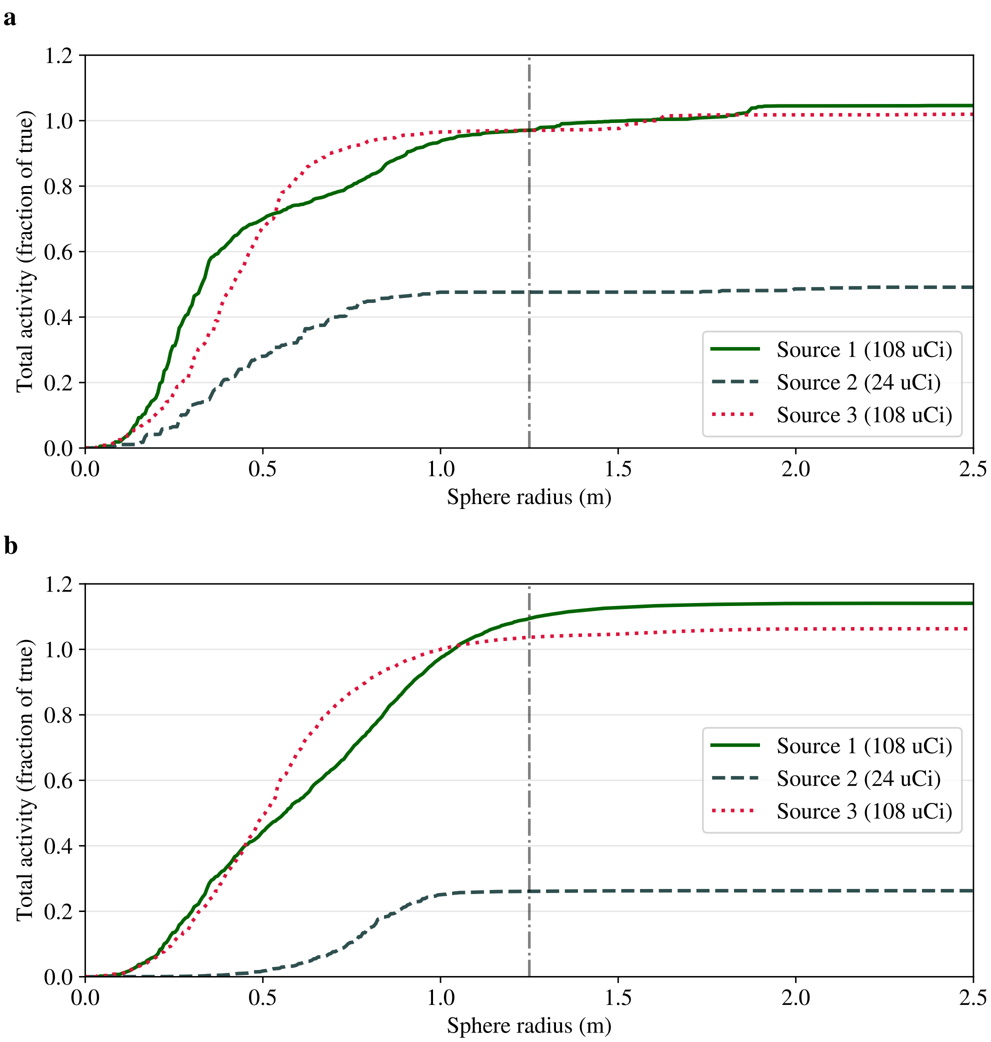

Figure S2. Total reconstructed activity estimation for distributed sources. Total reconstructed activity (as a fraction of the ground-truth activity) of the three source distributions in Fig. 3, calculated by summing the activity in voxels within a sphere around the center of the ground-truth source distributions. The summed activities are computed as a function of the spherical radius and a radius of 1.25 m (shown by the gray dashed-dotted line) was selected as the point at which approximately all three source activity reconstructions plateaued. Results are shown for Compton MAP-EM (**a**) and active coded mask MAP-EM (**b**) reconstructions.

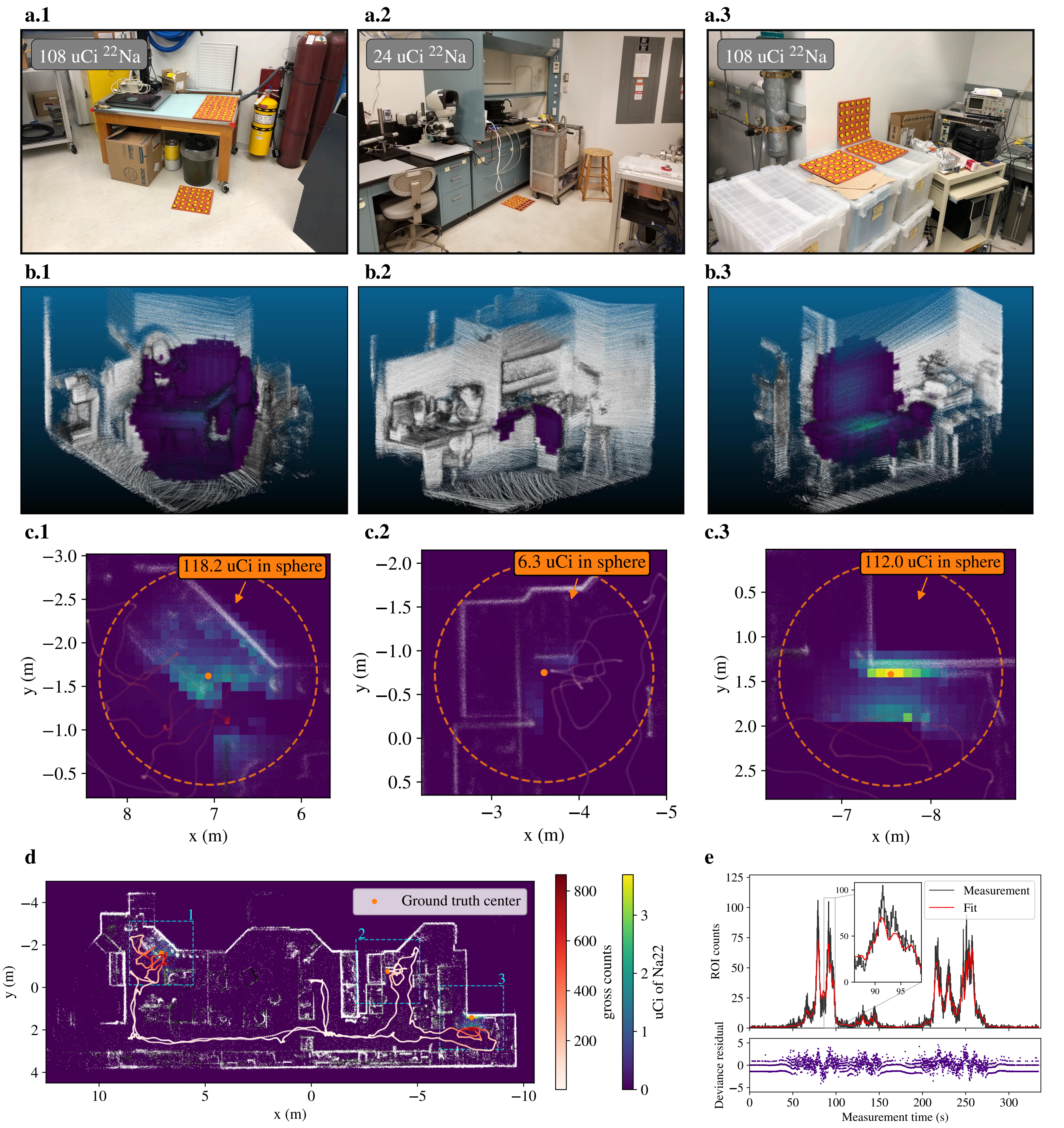

Figure S3. Distributed source reconstruction results. (**a**) Setup of the three distributed source distributions placed in a cluttered laboratory environment. (**b**) Cropped renderings of the 3D point clouds at the source locations, colorized by the quantified active coded mask MAP-EM reconstruction results with the bottom 1% of the reconstructed activity clipped to increase contrast with the point cloud. (**c**) Top-down projections of the reconstruction in the three source areas, with a 1.25 m sphere (in orange) around the center of the ground-truth sources to determine the reconstructed source activity. (**d**) Full top-down projection of the reconstruction result. (**e**) Forward projection of the reconstructed image overlaid on the measured data, summed across all detectors.

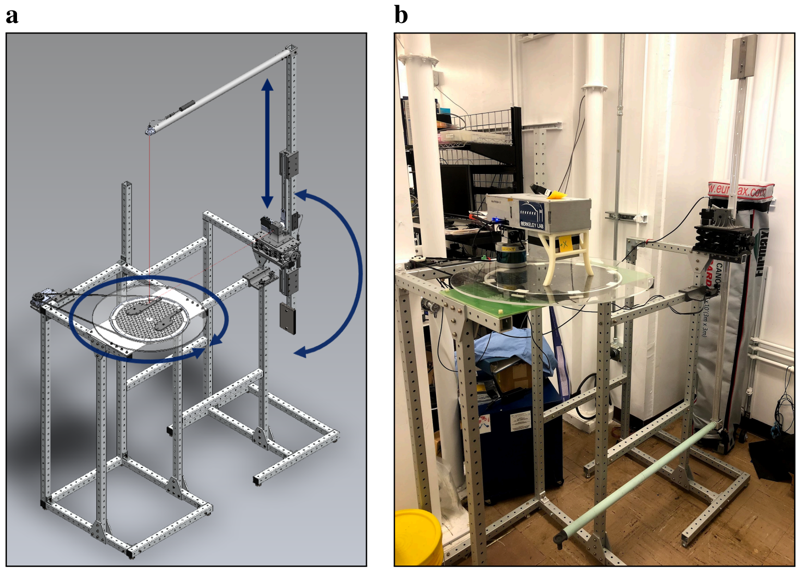

Figure S4. Detector response characterization setup. (**a**) Rendering of the 4π detector response scanner and concept of operation. (**b**) The MiniPRISM system on the prototype scanner in the laboratory.
